# Recent advances in endogenous neural stem/progenitor cell manipulation for spinal cord injury repair

**DOI:** 10.7150/thno.84133

**Published:** 2023-07-09

**Authors:** Jincheng Li, Wenqi Luo, Chunsheng Xiao, Jianhui Zhao, Chunyu Xiang, Wanguo Liu, Rui Gu

**Affiliations:** 1 Department of Orthopaedic Surgery, China-Japan Union Hospital of Jilin University, Changchun, 130033, People's Republic of China.; 2 Key Laboratory of Polymer Ecomaterials, Changchun Institute of Applied Chemistry, Chinese Academy of Sciences, Changchun 130022, People's Republic of China.

**Keywords:** Endogenous neural stem/progenitor cells, neurogenesis, spinal cord injury repair, combined treatment

## Abstract

Traumatic spinal cord injury (SCI) can cause severe neurological impairments. Clinically available treatments are quite limited, with unsatisfactory remediation effects. Residing endogenous neural stem/progenitor cells (eNSPCs) tend to differentiate towards astrocytes, leaving only a small fraction towards oligodendrocytes and even fewer towards neurons; this has been suggested as one of the reasons for the failure of autonomous neuronal regeneration. Thus, finding ways to recruit and facilitate the differentiation of eNSPCs towards neurons has been considered a promising strategy for the noninvasive and immune-compatible treatment of SCI. The present manuscript first introduces the responses of eNSPCs after exogenous interventions to boost endogenous neurogenesis in various SCI models. Then, we focus on state-of-art manipulation approaches that enhance the intrinsic neurogenesis capacity and reconstruct the hostile microenvironment, mainly consisting of pharmacological treatments, stem cell-derived exosome administration, gene therapy, functional scaffold implantation, inflammation regulation, and inhibitory element delineation. Facing the extremely complex situation of SCI, combined treatments are also highlighted to provide more clues for future relevant investigations.

## Introduction

Traumatic spinal cord injury (SCI) devastates the central nervous system (CNS) and inevitably leads to the disruption of neuronal circuits and neurological deficits. Since the self-renewal ability of the CNS following injury is very limited, SCI can induce many irreversible sequelae, such as paralysis, respiratory distress, and bladder dysfunction 1. According to epidemiological survey data, the global incidence rate of traumatic SCI is estimated at 23 cases per million, and patients endure heavy physical, financial, and psychological burdens 2. Currently, the available standard treatments for SCI include hemodynamic management, methylprednisolone pulse therapy, early surgical decompression, and rehabilitation, often preventing the continued loss of neurological function and complications but rarely achieving functional recovery 3.

The last three decades have witnessed a boost in exogenous cell transplantation (ECT) to promote SCI repair due to its significant paracrine effects and potential ability to replenish lost cells 4-6. The generally accepted amelioration mechanisms have been summarized, *i.e.*, neuroprotection, immunomodulation, axon regeneration, neuronal relay formation, and myelin regeneration 7. Early-phase clinical trials have shown that ECT is a generally feasible treatment that has achieved some encouraging results; however, its efficacy remains unproven, not to mention the operational, safety, and ethical concerns 8. More recently, an innovative neuronal reprogramming technology converting endogenous glial cells into functional neurons was developed by overexpressing neurogenic transcription factors (TFs) 9. Several reprogramming factors have been utilized and the feasibility of the method to generate neurons with distinct phenotypes has been validated, although reports with functional recovery effects at the behavioral level are rare 10, 11. Spinal cord resident endogenous neural stem/progenitor cells (eNSPCs) offer a safer and noninvasive solution for the treatment of SCI; through designated intervention, eNSPCs hold promise for neuronal differentiation and neural network construction, as well as oligodendrogenesis 12, 13.

The present review introduces the pathological changes of eNSPCs and the microenvironment they are immersed in after SCI and focuses on the current strategies developed to stimulate the neurogenesis of eNSPCs and relieve ambient inhibiting elements, particularly combinational treatments that have exhibited encouraging experimental performance. We expound on each individual approach based on their endogenous neurogenesis performance and summarize the enhancing and conflicting mechanisms of already tried combination therapies. Further endeavors should be devoted to addressing critical issues, such as the limited availability of eNSPCs and their restricted neuronal differentiation, to improve intrinsic nerve restoration and foster the SCI treatment process.

## Microenvironmental changes following SCI and their effects on eNSPCs

### Pathophysiological processes of SCI

Pathophysiologically, SCI can be divided into primary and secondary injuries, both of which can produce harsh microenvironments. In the primary injury phase, cell death, axonal membrane disruption, and breakdown of the brain-spinal cord barrier (BSCB) occur within a few minutes after the initial mechanical damage 14. Myelin-associated inhibitory molecules (MAIs), potent axon growth inhibitory molecules that are the breakdown products, *i.e.*, debris of the myelin sheaths, are immediately released, and the release process continues following the degenerative events caused by the injury 15. Blood-derived macrophages and local microglia react to the injury by releasing proinflammatory cytokines and chemokines, such as interleukin-1β (IL-1β), interleukin-6 (IL-6), and tumor necrosis factor-α (TNF-α), which further induce an overwhelming inflammatory response in the first few weeks post-SCI 16. In the secondary injury phase, ongoing necrosis of neurons and glia releases damage-associated molecular patterns (DAMPs) and further triggers the immune system. DAMPs engage pattern recognition receptors on inflammatory cells to activate tissue-resident macrophages and circulating neutrophils, resulting in the worsening of demyelination and exacerbation of tissue destruction 17. Ischemia and glutamate-mediated excitotoxicity disrupt ionic homeostasis and cause intracellular calcium dysregulation in both neuronal and glial cells. Continuing hemorrhage, increasing edema and inflammation lead to more severe damage, including the production of reactive oxygen species (ROS), lipid peroxidation, and nitric oxide production 16. At the middle to the chronic stage, microglia clear DAMPs, leaving behind a cystic cavity. A glial scar consisting of reactive astrocytes starts to form around the cyst 18.

### The origin of eNSPCs

Multipotent, self-renewing eNSPCs were first isolated from the adult mammalian spinal cord in 1996 by Weiss *et al.* 19. These cells can be grown as free-floating neurospheres and hold the capacity to differentiate into neurons, oligodendrocytes, and astrocytes. However, identifying the exact origin of these multipotent stem cells *in vivo* has been challenging. Three proliferative stem-cell-like cell types, namely, astrocytes (GFAP^+^/Sox9^+^/Cx30^+^), oligodendrocyte precursor cells (OPCs, NG2^+^/OLIG2^+^), and ependymal cells (EpCs, FoxJ1^+^), have been suggested as spinal cord stem cells 20, 21. Astrocytes can only self-renew to generate new astrocytes, and OPCs can only increase their rate of division and generate large numbers of remyelinating oligodendrocytes, which means that astrocytes and OPCs are not multipotent. EpCs, ciliated cells lining the brain ventricles and the spinal cord central canal, are key components of the ventricular-subventricular zone and the central canal stem cell niche. In the intact spinal cord, ependymal cells seldom divide; *in vitro*, they vigorously divide to produce astrocytes, oligodendrocytes, and neurons, evidencing their multipotency 22, 23. In the past two decades, the consensus that eNSPCs originate from EpCs in the spinal cord has gradually emerged.

EpCs are a heterogeneous group of cells. In rodents, the dorsal and ventral midline EpCs are characterized by a very long radial morphology 24. These cells maintain long filament-rich processes extending to the pia and are sometimes referred to as tanycytes. At the lateral level, EpCs are either cuboidal or show a radial morphology with a process ending on a vessel. Astrocytes contacting the central canal and cerebral fluid contacting neurons also exist in the EpC niche. One distinguishing feature of non-human primate EpCs compared with those in rodents is the presence of lateral EpCs with multiple ciliae. These cells do not express Nestin and do not proliferate. In comparison, dorsal and ventral cells are uniciliated or biciliated, express Nestin and/or GFAP, and proliferate 25. Human spinal cord ependymal cells appear similarly heterogeneous and undergo dramatic changes with age. In infants and teenagers, ependymal cells resemble those of adult mice and organize around the central canal, but in adults, this structure tends to collapse into an ependymal cell mass 26. As an origin for eNSPCs, multipotent EpCs exist in specific subgroups. One study identified a small subpopulation (8% of ependymal cells and 0.1% of all cells) marked by Troy and denoted as ependymal A (EpA) cells, that accounted for the *in vitro* stem cell potential in the adult spinal cord of mice 27. EpA cells were most abundant in the dorsal pole of the ependymal layer. Single-cell transcriptome analysis revealed a loss of ependymal cell gene expression programs as EpA cells gained signaling entropy and dedifferentiated to a stem-cell-like transcriptional state after an injury. Similarly, another study described a subgroup of EpCs in the lateral central canal (CC) that exhibited low expression of Sntn and an immature state, potentially indicating spinal cord stem cells compared with cells with high expression of Sntn. However, in the human spinal cord, most EpCs highly express Sntn correlating with the apparent lack of ependymal cell proliferation in the adult human spinal cord. Nevertheless, our understanding of the stem cell performance and heterogeneity of EpCs is still not very clear.

Recently, some studies have questioned the accuracy that eNSPCs originate from EpCs 28-31. Sofroniew's group found that EpCs did not contribute any component to the glial scar after a slight injury that did not damage the CC, and even after large damage that injured the CC, EpC-derived progeny remained local, did not migrate and contributed less than 2% cells of any kind 28. A study from He's group found that, once the SCI occurred, few Nestin^+^ (a marker of neural stem cells) cells were found in the CC but a large amount of Nestin^+^ cells already resided in the verge and fasciculus gracilis; these started to proliferate from the first day after SCI. Subsequently, BLBP (a marker of radial glial cells) and Nestin co-staining revealed that these Nestin^+^ cells were derived from radial glial cells in the parenchyma verge and fasciculus gracilis. This study provides a different viewpoint on the source of eNSPCs and suggests that radial glial cells are potential manipulation targets for endogenous neurogenesis 30. Dai's group found that pericytes contribute Nestin^+^ cells at the lesion core, and GAFP^+^ astrocytes contribute Nestin^+^ cells at the edge of the lesion 32. In addition, they also compared the Nestin^+^ cells inside and outside the CC and found that those outside the CC had higher activity, making them a potential source of eNSPCs 31.

### Microenvironmental changes and eNSPCs responses following SCI

The harsh environment includes high concentrations of the excitatory neurotransmitter glutamate, ROS, inflammatory factors, and myelin-associated inhibitors, *etc.* However, the effects of these microenvironment changes on eNSPCs are not quite clear 21. In *in vitro* cultures of spinal EpCs under a 500 uM glutamate level, EpCs proliferate significantly and differentiate into astrocytes. The specific mechanism may be as follows: glutamate-mediated calcium (Ca^2+^) influx through Ca^2+^-permeable alpha-amino-3-hydroxy-5-methyl-4-isoxazole propionic acid (AMPA) receptors (CP-AMPARs) in concert with Notch signaling increases the proliferation of EpCs via phosphorylated CREB and induces astrocytic cell fate specification through Hes1 upregulation 33. An in vivo study showed that positive allosteric modulation of AMPA receptors (AMPARs) subacutely after SCI increases EpCs proliferation, and astrogliogenesis also proved this viewpoint 34. Neurons and glial cells are sensitive to oxidative stress, which induces functional impairment or even death. However, neural stem/progenitor cells (NSPCs) exhibit unique responses, with low concentrations of ROS (2-4 µM) increasing the proliferation and survival of NSPCs 35. Medium concentrations (100 µM) promote neural differentiation and oligodendrocyte differentiation of NSPCs 36, while NSPCs only die when ROS concentrations reach up to 500 µM 37. These studies are still *in vitro* and whether they have the same effect in animal models remains to be seen. Necrosis of neurons and glia releases DAMPs including high mobility group box 1 (HMGB1), ATP, Ca^2+^, nuclear debris, *etc.* and further engages pattern recognition receptors on inflammatory cells to activate tissue-resident macrophages and circulating neutrophils, resulting in the worsening of demyelination and exacerbation of tissue destruction 17. In *in vitro* models, the addition of HMGB1 to an ependymal cell culture obtained from mouse spinal cord tissue promoted EpC proliferation and astrocyte differentiation; this process was inhibited by an HMGB1 antibody 38. Similarly, DAMPs, produced by repeatedly freeze-thawing a mixture of macrophages, promoted the differentiation of NSPCs from mouse brains into astrocytes *in vitro* 39. Inflammatory cytokines are also involved in regulating the fate of NSPCs. IL-6 promotes astrocyte differentiation of cultured NSPCs through the JAK/STAT pathway, and IL-6 receptor inhibitors inhibit the proliferation of reactive astrocytes in animal models 40. IL-1β inhibits proliferation and promotes apoptosis of cultured NSPCs through the SAPK/JNK pathway 41. MAIs are also factors that promote the astrocyte differentiation of eNSPCs and the details are explained in section 3.6. The influence of other components of the SCI microenvironment, such as acidity, ion overload, *etc.*, on the behavior of eNSPCs is yet to be determined. But in general, the post-SCI microenvironment promotes the proliferation and astrocyte differentiation of eNSPCs, thus forming a glial scar.

**Figure 1:**
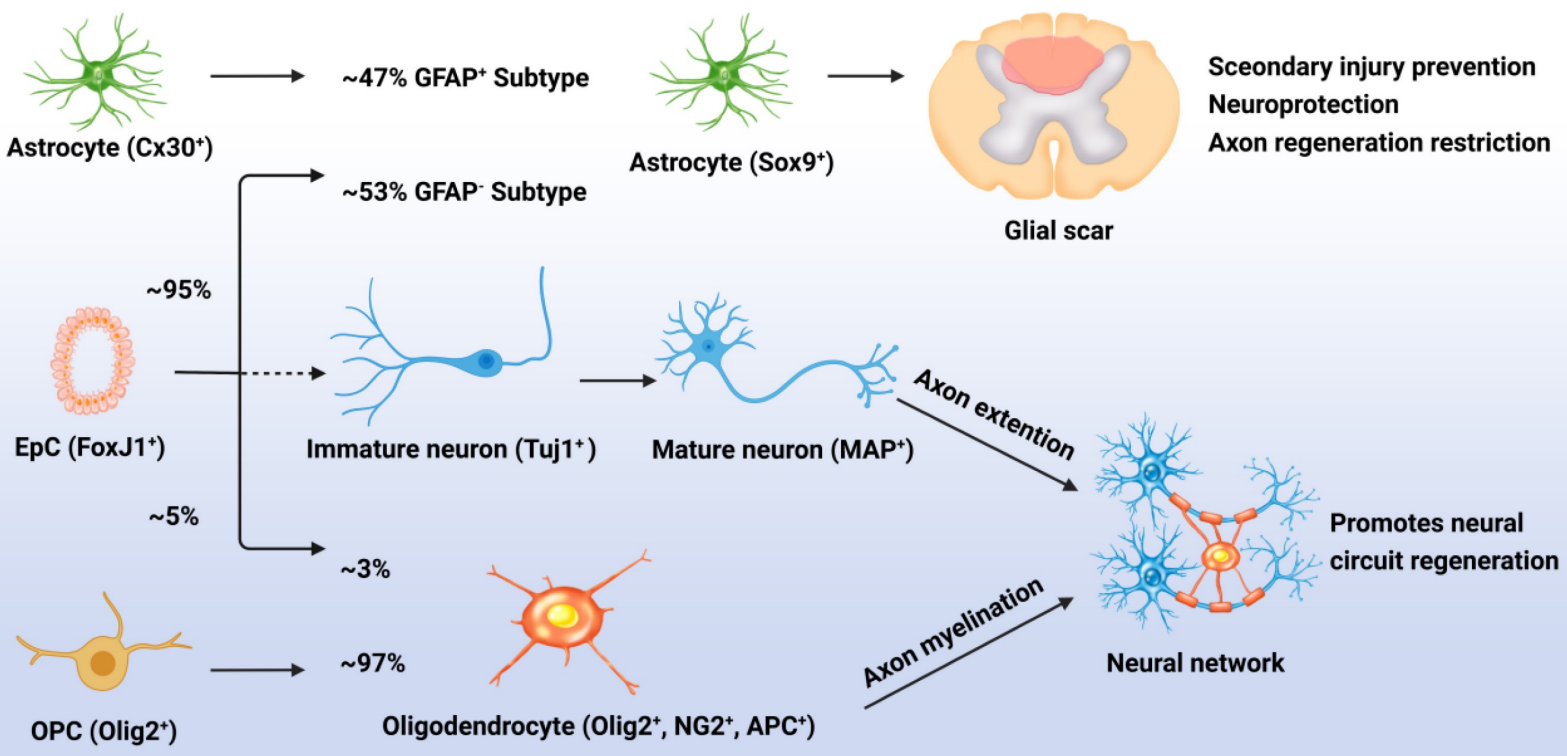
Responses of EpCs after SCI. Post SCI, 95% of the EpCs differentiated into astrocytes, accounting for 53% of newly generated astrocytes (GFAP^-^), with the remaining 47% of new astrocytes being derived from the proliferation of resident astrocytes (GFAP^+^). These cells promote glial scar formation which can prevent secondary injury but restrict neural regeneration. Less than 5% of the activated EpCs differentiated into myelin-forming oligodendrocytes, accounting for 3% of newly generated oligodendrocytes, with the remaining 97% of newly generated oligodendrocytes being derived from the differentiation of OPCs. Unfortunately, almost none of the EpCs differentiated into neurons which leads to the lossof neural circuit regeneration.

Although normally quiescent, eNSPCs are massively activated after injury, and the density of Nestin^+^ cells decreases with distance from the injury site 42. The primary differentiation process for these generated eNSPCs is astrocyte differentiation; results have shown that after injury, 95% of the EpCs differentiated into astrocytes, accounting for 53% of newly generated astrocytes (GFAP^-^), with the remaining 47% of new astrocytes being derived from the proliferation of resident astrocytes (GFAP^+^). Less than 5% of the activated EpCs differentiated into myelin-forming oligodendrocytes, and almost none differentiated into neurons 20, 43. Indeed, the adult eNSPC neurogenesis process has been defined as follows: active eNSPCs are recruited to the lesion/disease location, where they subsequently develop into immature neurons (Tuj1^+^) and then mature neurons (MAP^+^), and cultivate neural networks with host tissue, eventually leading to functional recovery. The key issue for neurogenesis is stimulating the neuronal differentiation of eNSPCs, which rarely occurs spontaneously after SCI (**Figure 1**) 23.

Redeployment of molecular pathways that are active during embryonic development but silent in the adult spinal cord facilitates successful spinal cord regeneration 44-47. Wnt, bone morphogenetic proteins (BMP), and Notch signaling are major regulators of embryonic neurogenesis, all of which are activated once trauma occurs 44. Previous studies have validated that the BMP and Notch pathways, together with the JAK/STAT and ERK/MAPK pathways, promote astrogliogenesis after SCI 47-50. Wnt/β-catenin signaling is an activated regulatory pathway that benefits neuronal differentiation 45, 51, however, Wnt mRNA expression is only markedly upregulated within the first week post-injury, principally resulting in the proliferation of eNSPCs 52. The underlying mechanism of the activation and subsequent neural differentiation of eNSPCs remains to be further clarified to promote neurogenesis.

## Promoting neurogenesis of eNSPCs located in the SCI niche

**Figure 2:**
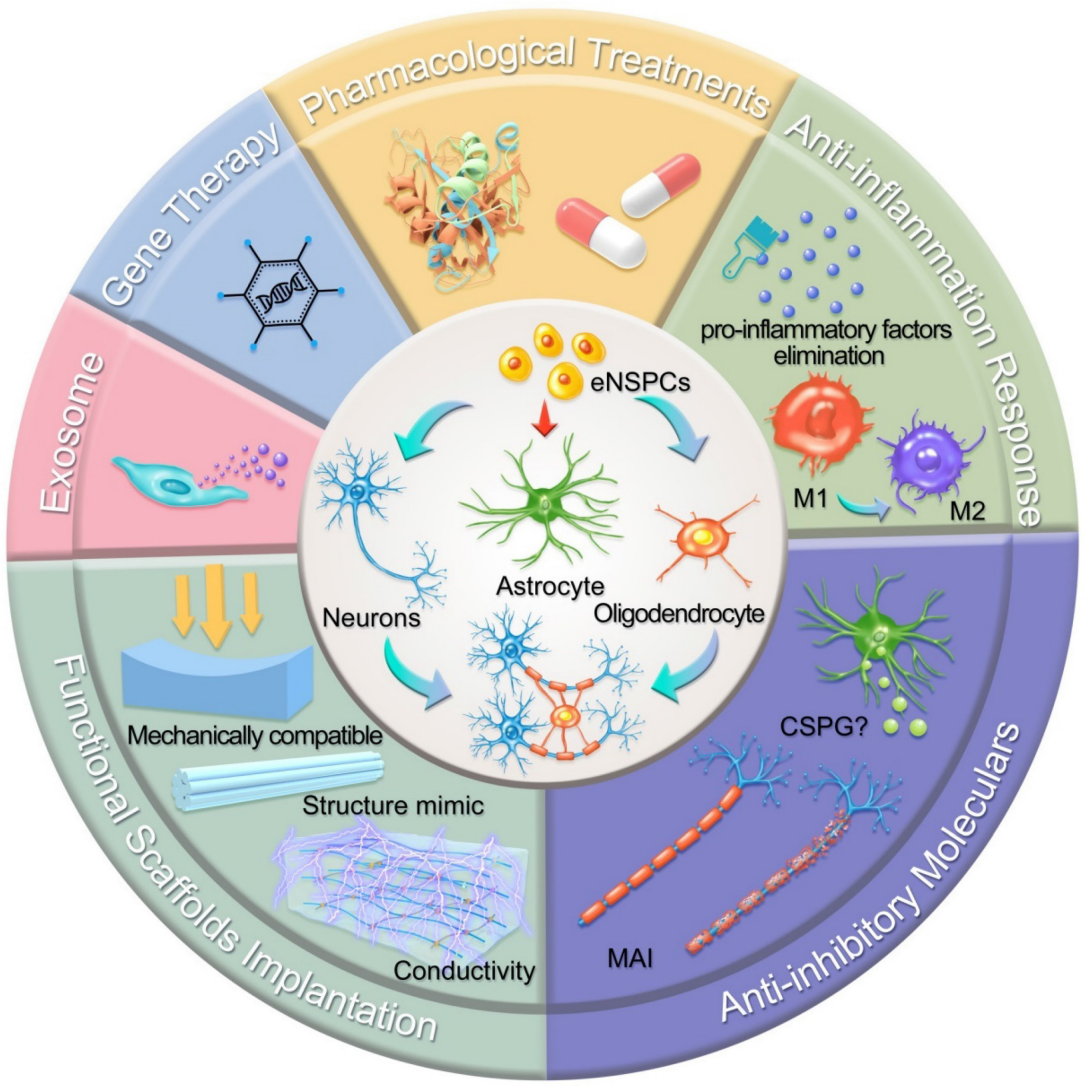
Schematic illustration of typical strategies modulating eNSPCs for the treatment of SCI. Pharmacological treatments, stem cell-derived exosome administration, gene therapy, and functional scaffold implantation can boost eNSPCs' intrinsic proliferation and differentiation capacity. Inflammatory regulation and inhibitory element delineation can remold the hostile microenvironment. All these strategies trigger eNSPCs to differentiate into neurons and oligodendrocytes but not astrocytes.

In this section, we highlight the efforts devoted to promoting neural circuit formation through manipulation of eNSPCs, mainly referring to the EpCs and neural stem/progenitor cells outside the central canal. According to two aspects, these efforts are aimed at boosting their intrinsic proliferation and differentiation capacity and remolding the hostile microenvironment. Strategies in allusion to the former aspect are categorized as pharmacological treatments, stem cell-derived exosome administration, gene therapy, and functional scaffold implantation, while methods aiming at the latter aspect consist of inflammatory regulation and inhibitory element delineation, as demonstrated in **Figure 2**.

### Pharmacological treatments

Many secreted biomolecules, mainly present in the nervous system and collectively called regeneration factors, such as neurotrophic factors, growth factors, chemokines, and hormones, can stimulate neuronal cell development and differentiation, and maintain their survival. Normally, the amount of regeneration factors in the damaged niche is too low to exert a positive effect on endogenous neurogenesis 53. Therefore, delivery of sufficient exogenous regeneration factors to the injury site is necessary for neural repair and functional recovery. Neurotrophic factors, as the most typical regeneration factors, are signaling proteins that bind to receptor tyrosine kinases located in NSPC membranes, *e.g.*, nerve growth factor (NGF) binds to TrkA, brain-derived neurotrophic factor (BDNF) binds to TrkB, and neurotrophin-3 (NT3) binds to TrkC, subsequently activating downstream signaling pathways that enhance neuronal survival, induce neurogenesis and synaptogenesis, alter the glial phenotype, and promote plasticity and axonal regrowth 54, 55.

In a long-span severe hemisection rat model, NGF embedded into nanofiber hydrogels composed of silk protein achieved scarless spinal cord repair and successfully restored motor function 56. One of the advantages of NGF is that neural/astroglial differentiation can be regulated by adjusting the immobilized amount to reconstruct the actual ratio of neural/astroglial cells in the normal spinal cord tissue. In 2015, Li's group reported that putting a biodegradable NT3-coupled chitosan into a 5-mm entirely transected and excised thoracic segment of a rat's spinal cord promoted new eNSPC neurogenesis and the nascent synaptic network formation through activating NT3-TrkC signaling 57. In a canine SCI model, the implantation of an NT-3/fibroin complex scaffold similarly led to significant neural regeneration. Similarly, another study transplanted NT-3 modified collagen scaffolds into SCI rhesus monkey models. A large number of neurons (Tuj-1^+^ and NeuN^+^) were detected within the lesion area in the LOCS+CBD-NT3 group and functional mature neurons like GABA^+^ and TH^+^ neurons were also found in the combinatorial group 58. BDNF can somewhat slow astrocyte differentiation while accelerating the differentiation of newborn and mature neurons 59. In contrast to NGF and NT3, BDNF directly contributes to the NSPC's oxidative defenses by boosting the activity of antioxidants, which is advantageous for neuronal survival 37. The inclusion of BDNF in 3D-printed collagen/chitosan scaffolds could help to further stimulate neural regeneration, prevent the occurrence of cavities and glial scar, and ameliorate locomotor function at the injury site 60. Ciliary neurotrophic factor (CNTF), a member of the hematopoietic superfamily of cytokines, can promote neuronal differentiation of NSPCs by binding CNTF receptor α and Glycoprotein 130 to recruit leukemic suppressor receptor β, thus activating the MAPK pathway 61. Implanting CNTF-doped sodium hyaluronate scaffolds into the lesion area resulted in the activation of EpCs, and a new connection between the newborn neurons and host tissue was observed 62.

Epidermal growth factor (EGF) and basic fibroblast growth factor (bFGF) are the two most frequently used growth factors. Previous mouse model results have indicated that EGF increases neurogenesis after SCI while reducing oxidative stress and apoptosis 63, 64. Yeoh and de Haan proposed that bFGF might drive NSPCs to develop into neurons as well as enhance the proliferation and division of NSPCs 65. Experiments showed that continuous intrathecal infusion of EGF and bFGF promoted eNSPC proliferation, migration to the injured area, and, to a certain extent, locomotor function recovery, but no newborn neurons were observed 66. Despite these positive findings, other research suggests that EGF may hamper the regeneration of the CNS by motivating BBB disruption and accelerating astrogliosis post-injury 67. Independently applied growth factors impose limited or even controversial impacts on neuronal regeneration.

Researchers have long been interested in unveiling the underlying facilitation mechanism of other regenerative factors during spinal cord healing processes. Substance P (SP) is a neuropeptide involved in cell proliferation and the synthesis of cytokines and growth factors in different kinds of cells 68, 69. SP induced robust eNSPC activation and promoted motor function recovery in SCI mice, the primary mechanism of which may be related to the Erk1/2 signaling pathway 70. A recent study demonstrated that erythropoietin (Epo), a glycoprotein hormone that promotes erythroid progenitor cell differentiation, could promote eNSPC neural differentiation and oligodendrocyte differentiation in the injured spinal cord, but with undetectable eNSPC proliferation 71. Notably, after SCI, both Epo and SP act as neuroprotective agents that enhance cell survival and anti-inflammatory capacity 72, 73. Stromal cell-derived factor-1α (SDF1α), a cytokine with a specific effect on eNSPC migration, is crucial for NSPC recruitment through interacting with its cognate receptor CXC chemokine receptor 4 74. It should be noted that in taking advantage of the strong migration promotion capacity of SDF1α, multifaceted strategies have been developed by combining SDF1α with other regeneration factors to create a new neural circuit in the lesion core 75, 76.

The short half-life of regenerative factors requires continuous infusion, which raises several issues: in particular, tissue damage at the infusion site and limited diffusion. Small molecular medicines can be potential candidates to replace regenerative factors in promoting endogenous neuronal regeneration, owing to their intrinsic advantages of easy accessibility, high stability, and beneficial process ability 12, 77. Tentative explorations have been made with traditional antitumor, anti-inflammatory, and anticonvulsive drugs to test their effects on NSPCs' neurogenesis. Paclitaxel (PTX or Taxol, an antitumor drug) 78, valproic acid (VPA, an anti-convulsive drug) 79, 80, and curcumin (an anti-inflammatory drug) 81 have all been verified to enhance neuronal differentiation through the Wnt/β-catenin signaling pathway, while metformin (an anti-diabetic drug) promotes the proliferation, self-renewal, and differentiation of NSPCs by activating the AMPK-aPKC-CBP pathway.

Liu *et al.* clarified that PTX regulated the neuronal differentiation of both cultured and endogenous spinal NSPCs, while NT3 promoted axonal extension and regeneration 78. In addition, NT3 was not as effective as PTX in promoting neuronal differentiation and regeneration of spinal cord NSPCs. In a canine SCI model, a PTX-modified collagen scaffold was implanted into the injured spinal cord, and a higher density of newborn neurons (Tuj-1^+^) and mature neurons (NeuN^+^) was observed in the lesion core compared with the control group. These results proved the ability of PTX to promote endogenous neurogenesis in large animals 82. VPA has long been used as an anticonvulsant and mood stabilizer, with its anticancer effects uncovered recently 83. In a SCI rat model, VPA-labeled chitosan nanoparticles (VA-CN) were constructed and administered, which efficiently benefited the reconstruction of tissue and recovery of locomotor functions 84. Results showed that VA-CN greatly increased NSPC proliferation and levels of neurotrophic factor expression after SCI, decreased the number of microglia and enhanced the differentiation of eNSPCs. Curcumin is used in some cultures to treat disorders linked to oxidative stress and inflammation, and previous cell-culture experiments have shown that curcumin encouraged the proliferation of embryonic cortical NSPCs and spinal cord neural progenitor cells (SC-NPCs) *via* the MAPK pathways 85. The impact of curcumin on SC-NPC was dose-dependent; that is, a lower dose (≤ 1 μM) of curcumin increased SC-NPC proliferation, whereas a higher dose (≥ 5 μM) had the opposite effect. A further study claimed that curcumin could promote the expression of eNSPCs in the injured spinal cord and reduce the activity of reactive astrogliosis and the lesion cavity 86.

### Stem cell-derived exosome administration

Exosomes, the smallest extracellular membranous vesicles (~30-150 nm), contain nucleic acids (*e.g.*, DNA, mRNA, noncoding RNA), proteins (*e.g.*, CD63/CD81/CD9, HSP60/HSP70/HSPA5/CCT2/HSP90, TSG101, AIP1/ALIX), and lipids (*e.g.*, cholesterol, phosphatidylserine, phosphatidylinositol), suggesting a critical role in cell-cell communication 87. NSPC-secreted exosomes (SC-Exos) can exert neurogenic, neurotrophic, anti-apoptotic, and anti-inflammatory effects while avoiding the limitations of the original stem cells, such as tumorigenicity, thrombogenicity, and immunogenicity 88. A retrospective study based on animal SCI models indicated that SC-Exos derived from different stem cells increased the expression of anti-inflammatory factors (IL-4, IL-10) and anti-apoptotic protein Bcl-2, and reduced the levels of pro-inflammatory factors (IL-1β, TNF-α) and apoptotic protein BAX, thus improving the motor function recovery 89.

Intravenous administration of human placental mesenchymal stem cell-derived exosomes (hpMSC-Exos) into completely transected animals promoted eNSPC proliferation and neurogenesis, demonstrating the ability of hpMSC-Exos to stimulate the recovery of motor and autonomic function after SCI 90. Dai's group provided the first evidence of the promotion capacity of eNSPC migration toward the lesion core by exosomes, even though the detailed mechanism remains ambiguous 91. Additionally, exosomes derived from transfected MSCs can serve as a vehicle for specific noncoding RNA delivery to treat SCI. MicroRNA (miR)-126 was efficiently transferred to exosomes derived from MSCs transfected with a miR-126 mimic, and the miR-126-loaded exosomes reduced the lesion volume and improved functional recovery by promoting neurogenesis and reducing cell apoptosis after SCI 92. Another promising study investigated the effect of exosomes purified from K^+^-Cl^-^-cotransporter (KCC2)-overexpressing bone marrow stem cells (BMSCs) (ExoKCC2) on SCI repair *in vivo*. Compared with BMSC-Exo, ExoKCC2 showed the advantages of neuron migration and differentiation promotion, resulting in better regeneration performance 93.

### Gene therapy

**Figure 3:**
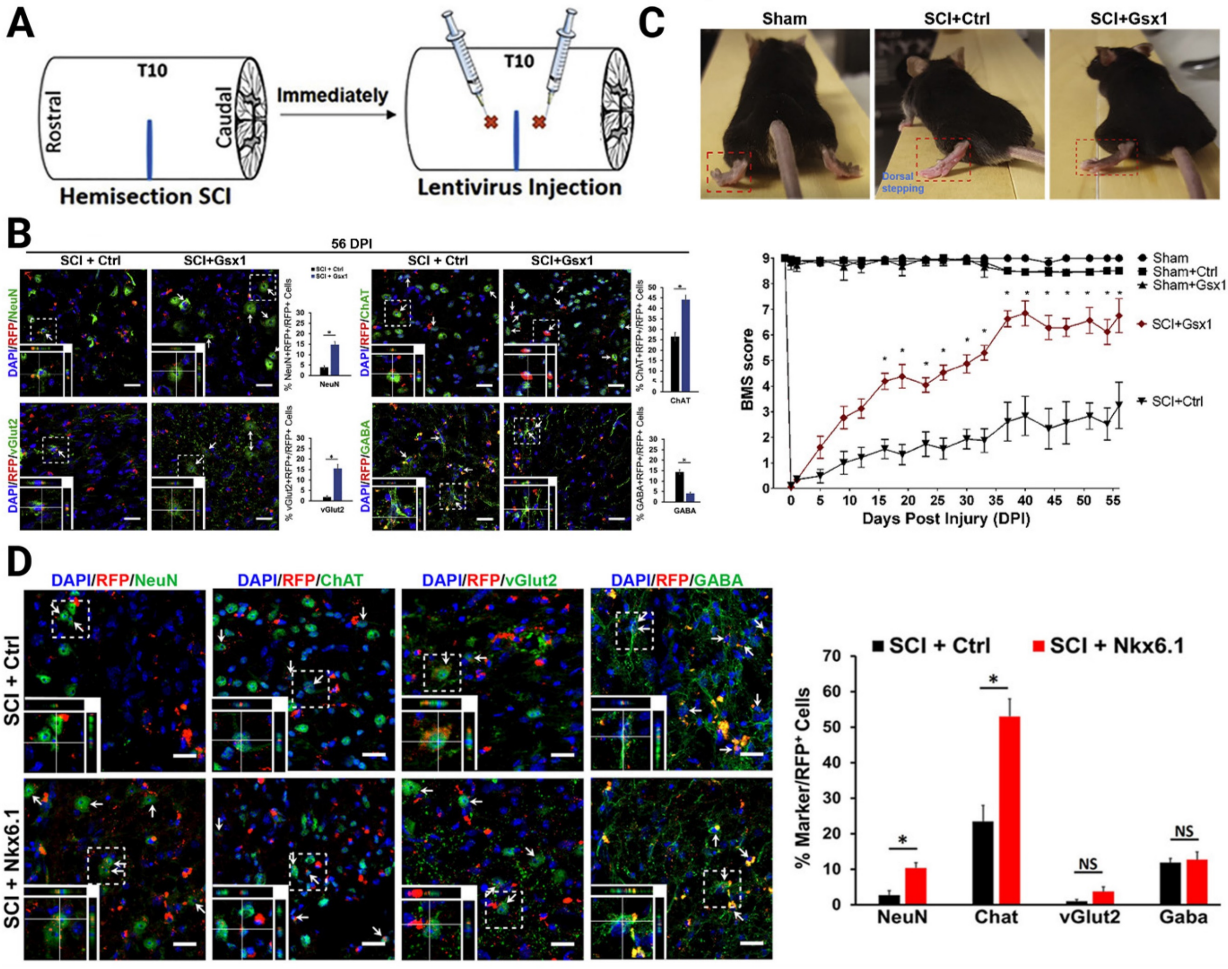
Lentivirus-mediated expression of neurogenic TFs increased neurogenesis of specific interneurons. (A) Lateral hemisection SCI was performed on 8- to 12-week-old mice at the T9-T10 level immediately followed by the injection of lentivirus encoding Gsx1 along with a RFP reporter (lenti-Gsx1-RFP). Lentivirus encoding, only the reporter RFP was used as a control (lenti-Ctrl-RFP). (B) Confocal images of sagittal sections of spinal cord tissues at 56 DPI show the expression of the viral reporter RFP and the mature neuron marker NeuN, glutamatergic neuron marker vGlut2, cholinergic neuron marker ChAT, and GABAergic neuron marker GABA, with quantification (n = 4). (C) Representative images of walking posture at 56 DPI and a plot of the BMS scores of the left hindlimb over 56 DPI. (D) Representative confocal images of the sagittal section of spinal cord tissue samples stained for the expression of mature neural markers: NeuN, ChAT, vGlut2, and GABA at 56 DPI and quantification of virally transduced cells co-labeled with a cell-specific marker. (A-C) Adapted with permission from 96, Copyright 2021 Elsevier. (D) Adapted with permission from 95, Copyright 2021 Elsevier.

Concerning the neurogenesis of eNSPCs after SCI, gene therapy allows investigators to modulate gene expression or introduce novel genes to boost intrinsic pro-regenerative cellular machinery, such as by increasing the expression of TFs and epigenetic modifications. Neurogenesis is most active during embryonic development, when numerous TFs participate in this process. It is generally believed that the decrease or absence of TFs in adult tissues results in poor neuronal regeneration 94-96. Based on the hypothesis that reactivation of these TFs in the injured adult spinal cord promotes cell proliferation and neural regeneration, Cai's group focused on two target genes, genomic screened homeobox 1 (Gsx1 or Gsh1) and NK6 homeobox 1 (Nkx6.1), which they had previously shown to bind to a Notch1 enhancer and regulate the expression of Notch1 during embryonic development of the brain and spinal cord 95-97. In particular, Gsx1 determines whether interneurons will become excitatory or inhibitory subtypes in the embryonic stage. After the spinal cord matures, its expression is often low or even undetectable. Nkx6.1 regulates NSPC specification and migration in the neural tube, as well as patterning throughout the development of the ventral CNS. The authors constructed a lentivirus delivery system to interrogate its effectiveness on neuronal regeneration (**Figure 3A**). Overexpression of Gsx1 promoted early-stage proliferation of eNSPCs, as well as promoted differentiation of eNSPCs into glutamatergic and cholinergic interneurons, and inhibited differentiation into GABAergic interneurons (**Figure 3B**). By affecting interneuronal subsets, it restored excitatory/inhibitory homeostasis in the injured spinal cord and ultimately promoted functional recovery 96 (**Figure 3C**). The overexpression of Nkx6.1 could also promote the differentiation of spinal eNSPCs into cholinergic interneurons, although the functional restoration effect was limited 95 (**Figure 3D**).

Sox11, a member of the SoxC subgroup, is primarily engaged in neural development and organogenesis at the fetal stage but is missing in adult spinal cord tissues. The overexpression of SOX11 causes neuronal differentiation in the adult subgranular zone of the brain by upregulating the immature neuronal marker doublecortin and the neuronal microtubule-associated protein MAP2AB. It has also been shown that overexpression of Sox11 in human glioma-initiating cells suppresses tumorigenicity by inducing neuronal differentiation. The overexpression of Sox11 leveraging a lentiviral vector containing the Sox11 gene in SCI mice contributed to the migration and neuronal differentiation of eNSPCs; however, the recovery of motor function was not significant, which partially resulted from the low transfection efficiency and lack of specific differentiation 94. A remarkable finding by Llorens-Bobadilla *et al.* uncovered the latent potential of EpCs to replace significant numbers of destroyed oligodendrocytes in a contused spinal cord 98. Integrating multimodal single-cell analysis, the authors found that chromatin regions with binding patterns for SOX10 and the oligodendrocyte transcription factor 2 (OLIG2) were accessible even when the TFs were not expressed. The accessibility of OLIG2 binding sites was significantly enhanced and the conductance velocity in spared axons above the damage site was improved when OLIG2 overexpression was induced in EpCs *in vivo*. However, in OLG2-overexpressed mice, motor-functional recovery was still not superior. Currently, combined treatment is needed.

The transcription analysis of some nonmammalian vertebrates with vigorous regeneration capacity after injury provides an alternative strategy to inspire gene treatment for SCI in allusion to eNSPCs. Natsume's group elucidated that Neurod4 (a basic-helix-loop-helix TF) was the most promising TF to exert neural regeneration after SCI in mammals. They developed a unique pseudotyped retrovirus that can selectively infect mitotic (activated) NSPCs derived from EpCs after SCI. The overexpression of Neurod4 induced the differentiation of eNSPCs into neurons with regenerative axons and inhibited the glial lineage of scar formation 99.

Epigenetic modifications play an important role in adult neurogenesis. Injury treatments based on epigenetic modification mechanisms often target intracellular regulators rather than fragments of the genome, such as noncoding RNAs. Experimental results have suggested the possibility of eNSPC manipulation through the handling of various miRs with positive outcomes on functional recovery after SCI 100-102.

### Functional scaffold implantation

The scaffolds commonly used in SCI repair can be divided into injectable hydrogel scaffolds and prefabricated implantable scaffolds. Both synthetic biomaterials 103 (*i.e.*, poly(e-caprolactone) (PCL), poly (lactic acid) (PLA), poly (lactic-co-glycolic acid) (PLGA)) and naturally extracted biomaterials 104 (*i.e.*, chitosan, alginate, agarose, hyaluronic acid (HA), collagen, gelatin, fibronectin) have been developed as high-quality scaffolds, which act as bridges for neural parenchymal cell adhesion and growth. One of the fundamental roles of scaffolds is to provide mechanical support for cells that replenish the damaged extracellular matrix (ECM), thus promoting constructive and functional tissue repair 105. Moreover, stem cells can modify their behavior by transforming the ambient altered physical cues into biochemical signals *via* mechanical sensors, therefore, the properties of the scaffolds (*i.e.*, stiffness, microstructures, and conductivity) can be deliberately modulated to impose a positive influence on eNSPC differentiation and neuronal neurite growth 106.

**Figure 4:**
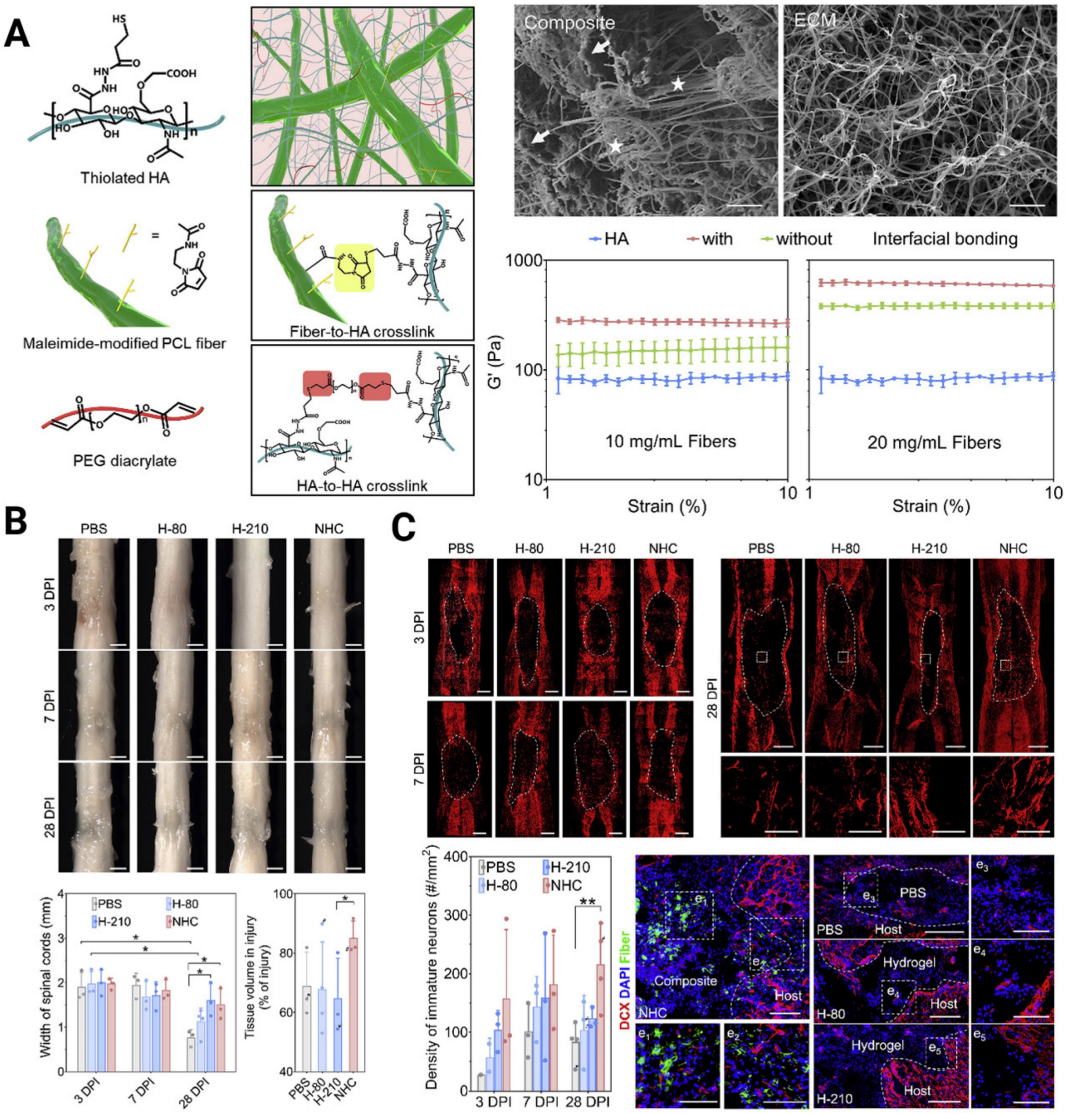
(A) Engineering a nanofiber-hydrogel composite with interfacial bonding between the fiber surface and hydrogel network. Left column: Schematic of the synthesis and structure of the PCL nanofiber-HA hydrogel composite (NHC). Right column: SEM images of the NHC (left) and porcine native spinal cord ECM (right), and the oscillatory strain sweep tests of the HA hydrogel and composites with and without interfacial bonding. (B) The NHC limits the collapse of the contused spinal cord and facilitates tissue formation in the injury. (C) Upper panel: The NHC stimulated neurogenesis in the injury. Microphotographs showing immature neurons stained with antibodies against βIII-tubulin and around the injury in representative horizontal sections of each treatment group at 3 (a), 7 (b), and 28 (c) days post-injury (dpi) and a representative area for each group at 28 dpi shown in high magnification. Bottom panel: Bar graph showing the average density of immature neurons per mm^2^ in the injury for each experimental group at 3, 7, and 28 dpi, and the microphotographs showing neural precursor cells stained with antibodies against doublecortin (DCX, red) in and around the injury in horizontal sections of each treatment group at 28 dpi. Adapted with permission from 112, Copyright 2020 Elsevier.

A crucial design criterion for the development of artificial scaffolds exists in effectively mimicking the 3D soft mechanical signature of native spinal cord nervous tissue (with a shear storage modulus on the order of 10^2^ Pa 107). In fact, both 2D and 3D *in vitro* studies have verified that NSPCs tend to differentiate toward astrocytes on harder surfaces (> 10^3^ Pa) and toward neurons on softer surfaces (<10^3^ Pa) 108-110. Chen *et al.* constructed a soft bionic scaffold with electrospun photocross-linked gelatin methacryloyl (GelMA) hydrogel fibers, which fostered the differentiation of eNSPCs into neuronal cells, prevented the growth of glial scar tissue, and stimulated angiogenesis 111. Consistent tendency results showed that neurons in softer GelMA fibers (~0.8 kPa) were found to be more prevalent than those in firmer gelatin fibers (~1.6 kPa), whereas astrocytes and glial scars were less prevalent. Another intriguing finding of their work is that the fabricated aligned GelMA fibrous bundle facilitated NSPC migration and guided axonal extension. Aside from aligning cells and directing cell migration, topographical cues, such as grooves, ridges, and pores, can affect stem cell fate. A unique nanofiber-hydrogel composite (NHC), created by Mao's team, imitates the mechanical characteristics and micro-architecture of a soft tissue matrix. The NHC is made up of a HA hydrogel phase that has undergone thiol modification and is covalently coupled to fragmented, electrospun PCL fibers and can facilitate the infiltration of endogenous cells 112 (**Figure 4A**). They validated the ability of the NHC to support the differentiation of eNSPCs into immature neurons and promote the shift toward a pro-regenerative macrophage population, angiogenesis, neurogenesis, and axon presence (**Figure 4B-C**). In large animals, this ability has been already tested. Wang's group created a soft stiffness and aligned nanofiber structure hydrogel to treat SCI canines, and the nerve regeneration rapidly crossed the lesion within 4 weeks 113.

Another feature of native spinal cord tissue worth noting is its high conductivity of ~8-130 S/cm 114, 115. Bioscaffolds with conductivity similar to that of nerve tissue should favor electrical signal transduction to neurons, and augment neural activity and neural-oriented differentiation by enhancing cell-cell contact 116. It has been revealed that electroactive biomaterials strengthen the weak local electric fields created by cell membranes, thereby manifesting transmembrane voltage gradients to influence ion influx across the cell membrane 106. However, developing multifunctional biomaterials that provide favorable physical and electrical cues simultaneously remains a challenge since conventional electronic materials are substantially stiffer than neural tissue. Incorporating conductive elements 106, 117, *e.g.*, conductive polymers, metals or metal-based nanomaterials, and carbon-based materials into established bioscaffolds, provides a solution to avoid a mechanical mismatch with the implanted site and at least partially mitigates their cytotoxicity. A novel and facial injectable hydrogel composed of agarose, gelatin, and polypyrrole (Aga/Gel/PPy) with optimal mechanical strength (1470 Pa) and conductivity (2 × 10^-3^ S/cm) was created, and it offered a biocompatible milieu for fostering endogenous neurogenesis 118. Further investigation using RNA sequencing analysis revealed that the Aga/Gel/PPy hydrogel drastically altered the expression of genes related to neurogenesis by activating intracellular Ca^2+^ signaling cascades.

**Figure 5:**
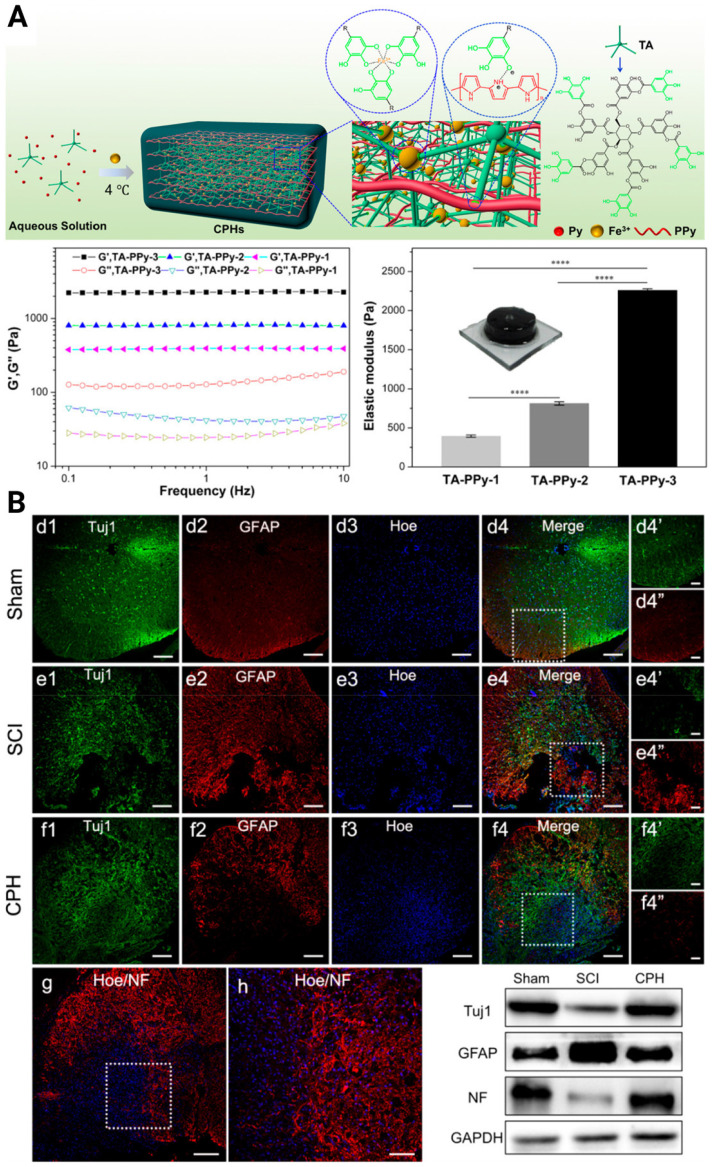
(A) Illustration of the fabrication of a cross-linked CPH following the addition of TA, Py, and Fe^3+^, as well as the physical and electrical properties of CPHs with different TA concentrations. (B) Upper three panels: Immunohistofluorescence images of transverse spinal cord sections obtained from animals in the sham, SCI, and hydrogel groups at 6 weeks. Bottom panel: NF staining of the regenerated nerve fibers in the hydrogel group, and the Western blot analysis of the spinal cord protein extracts showing the Tuj1, GFAP, and NF protein bands in the sham, SCI, and hydrogel groups. Adapted with permission from 121, Copyright 2018 American Chemical Society.

To pursue better tissue remodeling performance, other practical properties were integrated into the composites, such as self-healing and biodegradability. Luo *et al.* reported a natural ECM biopolymer (chondroitin sulfate and gelatin)-based hydrogel containing Ppy that possessed both favorable mechanical (~928 Pa) and conductive properties (4.49 × 10^-3^ S/cm) and exhibited shear-thinning and self-healing abilities 119. By triggering the PI3K/AKT and MEK/ERK pathways, and inducing myelinated axon regeneration, the electroconductive ECM hydrogel activated eNSPC neurogenesis and significantly restored locomotor function. Considering that conventional conductive phase-incorporated materials, *e.g.*, Ppy, polyaniline, carbon nanotubes, graphene, and gold nanowires, are nondegradable and that the residuals remain in the body as exogenous matter, conductive and biodegradable germanium phosphide nanosheets have been doped into an adhesive hyaluronic acid-graft-dopamine hydrogel (HA-DA/Gep@PDA) 120. Immunostaining results displayed the spatial distribution of Tuj-1 labeled neurons and GFAP-labeled astrocytes in the SCI lesion center, and the HA-DA/Gep@PDA treated groups exhibited the highest density of neurons and the lowest density of astrocytes. They speculated that the hybrid matrix explored could alleviate the local inflammation by consuming ROS and enhance angiogenesis by increasing the expression of positive regulators (MMP-2 and bFGF). Despite these achievements, scaffolds with conductivity similar to or higher than that of native spinal cord tissue have rarely been reported. In 2018, Lei Zhou et al created biocompatible conducting polymer hydrogels (CPHs) that had high conductivities (0.05-0.18 S/cm) and suitable mechanical characteristics (0.3-2.2 kPa) 121 (**Figure 5A**). After treatment with CPHs, Tuj1^+^ nerve tissue replaced the fibrotic scarring originally filling the large cystic cavities in SCI animals, and Nestin^+^ NSPCs were simultaneously seen in the affected lesion region, indicating the occurrence of endogenous neurogenesis (**Figure 5B**).

### Inflammatory regulation

As described in Section 2, the ambient overreacting inflammatory response during secondary injury cascades hampers eNSPC neurogenesis by inhibiting eNSPC proliferation and/or inducing eNSPC differentiation toward astrocytes. Drugs aimed at pro-inflammatory elements, including nonsteroidal anti-inflammatory drugs (NSAIDs), minocycline, cyclosporine A, and the corticosteroid methylprednisolone, have been employed to relieve inflammatory symptoms. Intrathecal/local injection 122 and implantable hydrogels 123 were commonly used to bypass the BSCB. Water-soluble selenium doped carbon quantum dots (Se-CQDs) synthesized with L-selenocystine with an excellent ROS scavenging ability were developed by our team, and effectively shielded both astrocytes (N2a cells) and neurons (PC 12 cells) against H_2_O_2_-induced damage *in vitro*. Compared with the saline-treated group, the Se-CQD-treated group achieved a significant decrease in cleaved caspase-3, Bax, and caspase-9 expression levels, and an increase in Bcl-2 expression levels. Moreover, the treatment group regained spontaneous urination faster and got higher BBB scores 124. Due to the existence of the BSCB, systemically administered anti-inflammatory drugs can hardly be delivered to the lesion core 125. The popularity of drug carriers, such as nanoparticles 126, 127 and liposomes 128, provides new resolutions for effective targeted delivery. Our recent work encapsulated selenium nanoparticles byepigallocatechin-3-gallate (EGCG-Se NPs), which not only endowed the nanoparticles with excellent solubility and biocompatibility but also manifested more potent antioxidant and anti-inflammatory properties. Intravenous injection of EGCG-Se NPs in a rat model of SCI exerted significant effects on locomotor function recovery 129. Another creative endeavor to realize noninvasive drug delivery for SCI repair that our group attempted was developing minocycline-loaded poly(α-lipoic acid)-methylprednisolone prodrug nanoparticles (MC-PαLA-MP NPs). The systemically administered MC-PαLA-MP NPs could rapidly accumulate in the spinal cord within 72h with a favorable drug release ability, which led to reduced inflammation in the microenvironment 130.

Manipulation of microglia/macrophage polarization serves as the principal method to achieve immune microenvironment modulation from the cellular perspective. *In vitro*, studies have demonstrated that an M1 conditioned medium could promote EpC proliferation while inhibiting neural differentiation at least partially by activating Sox2 expression (the TNFa-MAPK-Sox2 signaling pathway) 131, whereas an M2 conditioned medium could favor EpC differentiation toward mature neurons by upregulating Sirtuin2 expression (the BDNF/TRKB-MEK/ERK signaling pathway) 132.

The subsequent *in vivo* results further confirmed that depletion of M1 microglia/macrophages led to an increase in newborn neurons differentiated from eNSPCs by adding a colony stimulating factor 1 receptor inhibitor to the injured area 133. The M1/M2 polarization balance can also be regulated through gene regulation of microglia/macrophages. Rictor has previously been identified as an essential and unique component of the kinase mammalian target of rapamycin 2 (mTORC2) that directly activates mTORC2 function to contribute to axon growth. The overexpression of Rictor contributes to the M2 polarization of microglia/macrophages and the scavenging of proinflammatory factors 134. Protective autoimmunity of the body can also be exploited to modulate the immuno-microenvironment. A91, a peptide derived from the immunogenic sequence (87-99) of myelin-basic protein (MBP), exerts its anti-inflammatory effects by inhibiting lipid peroxidation, downregulating inducible nitric oxide synthase gene expression, reducing apoptosis, and inducing activation of Th2-lymphocytes that promote an M2 macrophage phenotype 135. A91 immunization increased anti-inflammatory cytokines and neurotrophic factors and decreased proinflammatory cytokines, suggesting the potential for motor and sensory recovery improvement in the chronic stage of SCI.

Traditionally, chondroitin sulfate proteoglycans (CSPGs) have been the main component of glial scars and are thought to inhibit axon growth after SCI 136, 137. In addition, CSPGs have a regulatory effect on the inflammatory response after SCI. CSPGs activate immune cells, exacerbating the inflammatory response of tissues through various mechanisms: interaction with immunological receptors on immune cells, such as TLR-2, TLR-6, CD44, LAR, and PTP σ receptors which leads to the activation of neuroinflammation; combination with inflammatory factors to form stable complexes, continuously activating receptors; and promotion of matrix metalloenzyme activity, which facilitates matrix digestion; and promotes inflammatory cell infiltration 138. Therefore, clearing CSPGs is a promising treatment method. Chondroitinase ABC (chABC) is a bacterial enzyme that through catabolism of CSPG glycosaminoglycan (GAG) side chains has been shown to increase axon regeneration and functional recovery following SCI 136, 137. Delivering chABC to the injured area, especially using biomaterials to locally and in a controlled way release chABC, reduces the deposition of CSPGs, the formation of glial scars, and improves the injured microenvironment, so that more eNSPCs can be distributed in the injured core to increase nerve regeneration. However, excessive clearing of CSPGs to suppress the inflammatory response is not a good approach, as CSPGs participate in the formation of glial scars, which limit the spread of inflammation and reduce secondary tissue damage. As more CSPGs are digested, more inflammatory cells can infiltrate the injury site; CSPGs play a role in limiting inflammation 139. The delicate CSPG balance thus poses a challenge for achieving the best functional recovery. Based on this mechanism, He's group doped intracellular sigma peptide (ISP) and intracellular LAR peptide (ILP), which antagonize LAR and PTPσ receptors, into a functional self-assembling peptide (F-SAP) hydrogel to cut the inflammatory signal caused by CSPGs 139. This strategy not only retained CSPGs but also inhibited the activation of the CSPG-mediated inflammatory response, which led to a permitted niche for eNSPCs neurogenesis.

It is worth noting that many implantable scaffold biomaterials, such as gelatin 133, 140, 141, chitosan, hyaluronic acid, decellularized extracellular matrices 142, 143, and synthetic polymers (*i.e.*, polylactic-co-glycolic acid) 103, have exhibited anti-inflammatory properties. Cheng's group recently developed a Mg/Al layered double hydroxide (LDH, a kind of clay with anionic and anion exchange properties) nanocomposite, which showed significant effects in promoting the M2 polarization of microglia/macrophages. The underlying mechanism was investigated in depth, and transforming growth factor-β receptor 2 (TGFBR2) was identified as the target receptor of LDH. The expression of Smad2/3 was downregulated by targeting TGFBR2, which raised the expression of TNF-α and IL-10, providing a favorable immunological milieu for the proliferation and functional differentiation of eNSPCs to enable neurogenesis 144.

### Inhibitory element delineation

Aside from neuroinflammatory conditions, the failure of neuronal regeneration after SCI is also attributed to inhibitory components, mainly MAIs and CSPGs, located in the impaired niche 3, 18. The debris of the disrupted myelin sheath of oligodendrocytes contains Nogo-A, MBP, myelin-associated glycoprotein (MAG), oligodendrocyte-myelin glycoprotein (OMgp), and some semaphorins and ephrins 105, 145. MAIs show a negative effect on eNSPC proliferation and neuronal differentiation. It has been reported that the Nogo-66 receptor 1(Ngr1) is the membrane receptor for multiple MAIs, including Nogo-A, OMG, and MAG, and that blocking or inhibiting Ngr1 can promote neuronal cell production and functional recovery after SCI 146-148. Simultaneously administering of PKA and ATP can phosphorylate NgR1 and impede the binding between MAIs and NgR1, thus increasing eNSPC proliferation 148.

**Figure 6:**
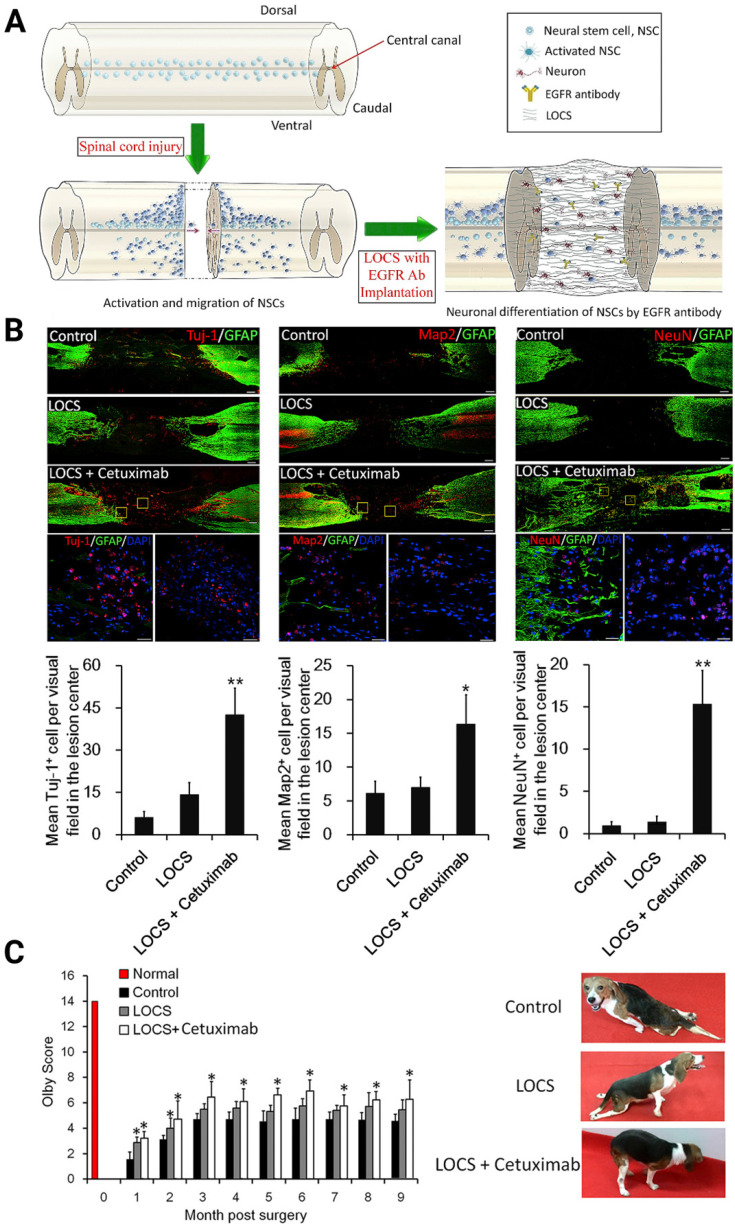
(A) Schematic of injury-induced activation and migration of NSPCs and cetuximab-promoted neuronal differentiation of NSPCs. Endogenous NSPCs remain quiescent in the intact spinal cord, but severe SCI can activate NSPCs and induce their migration into the lesion site. Blocking EGF signaling can promote the injury-activated NSPCs to differentiate into neurons. These newborn neurons can then build neuronal relays to bridge injury gaps and lead to functional recovery after SCI. (B) Endogenous neurogenesis and CSPG deposition in lesion sites of dogs with long-term T8 removal injury. Upper panels: Tuj-1-positive newborn neurons, MAP2^+^ mature neurons, and NeuN^+^ cells in the lesion sites of dogs in the control, scaffolds implantation and cetuximab-modified scaffolds implantation (LOCS+Cetuximab) groups at 9 months after injury. Bottom panels: enlargements of areas indicated in AeC (yellow boxes), and quantification of newborn neurons, mature neurons, and neuronal nuclei in lesion centers of dogs among each group. (C) Locomotion recovery, assessed with Olby scores, at each time point examined. Adapted with permission from 42, Copyright 2017 Elsevier.

Several methods that employ antagonists 42, 149, blockers 64, 78, or gene therapies to target relevant downstream signaling pathways, including mTOR/STAT 146, EGFR-ERK-TRIM32 64, and p38-MAPK 150, were then developed and comprehensively reviewed by Dai's group for rescuing neurogenesis inhibition by MAIs 151-153. For instance, Dai's laboratory studied the effect of Cetuximab, an EGFR inhibitor, functionalized linear porous collagen scaffold (LOCS) implanted into SCI areas (**Figure 6A**). They obtained an activation timeline and the pattern of eNSPCs following acute complete T8 spinal cord removal. The authors observed similar restoration manners in SCI rats and dogs: Tuj-1, Map2, and NeuN-positive neuronal cells were all more prevalent in the LOCS+Cetuximab group 42 (**Figure 6B-C).**

The other inhibitory components, CSPGs, are proteoglycans consisting of a core protein and glycosaminoglycan side chains secreted by active astrocytes 145, 154. Studies aimed at elucidating the effect of CSPGs on eNSPC neurogenesis are limited thus far. Recently, Soheila Karimi-Abdolrezaee's group found that CSPGs impede neurogenesis of transplanted NSPCs *in vitro* and *in vivo*; they demonstrated that the CSPG/LAR/PTPσ axis suppressed neuronal differentiation at least partially by blocking the Wnt/β-Catenin pathway 155. The addition of specific inhibitory peptides of LAR and PTPσ can reverse the inhibitory effect of CSPGs on NSPCs. Based on these results, investigating the impact of CSPGs on eNSPC neurogenesis is highly recommended, and identifying other inhibitory microenvironmental elements can be beneficial.

**Figure 7:**
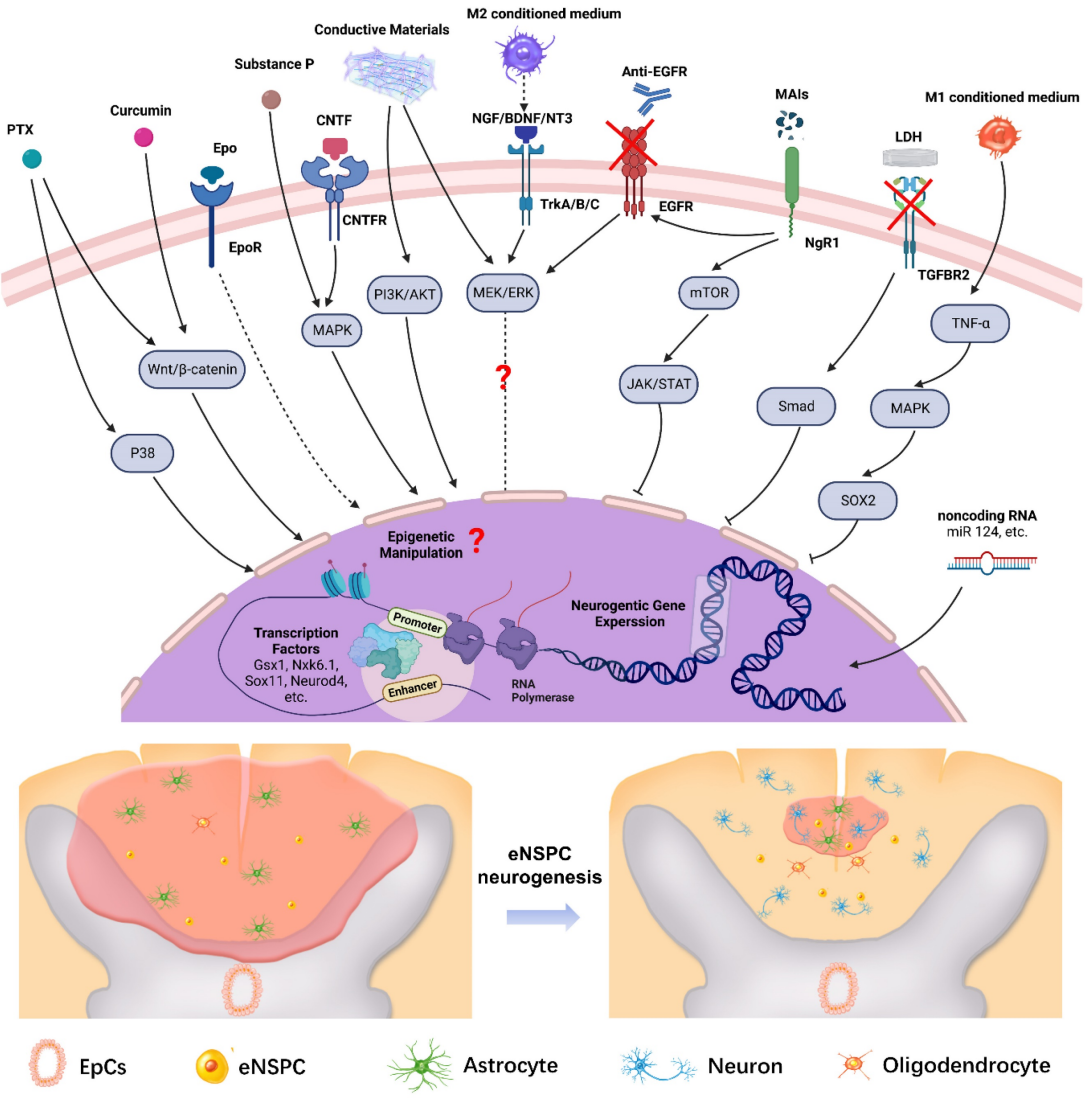
Illustration of the multilayered extrinsic and intrinsic mechanisms of regulating eNSPCs behavior post SCI. Delivering extrinsic niche signals (*e.g.* PTX, Curcumin, Epo, Substance P, CNTF, *etc.*) activates intracellular signaling pathways that induce changes in neurogenic gene expression. In addition, manipulating intrinsic transcriptional programs (*e.g.* transcription factors, epigenetic information, non-coding RNA, *etc.*) can direct neurogenic gene expression in eNSPCs. These strategies trigger eNSPC neurogenesis which leads to neural connection reconstruction and glial scar reduction. Arrows with dotted lines mean some steps of the signal pathway are omitted. Created with BioRender.com.

Effective therapy relies on dynamic operations of the cell-intrinsic (TFs, non-coding RNAs) and extrinsic regulatory networks (regeneration factors, drugs, MAIs, and inflammatory factors). **Figure 7** shows a summary scheme of the multilayered mechanisms that are involved in the neural induction of eNSPCs or the amelioration of the inhibitory microenvironment for endogenous neurogenesis. The extracellular components trigger intracellular signaling cascades, which affect gene expression or cell function directly. The critical molecular signaling pathways in embryonic or adult hippocampus neurogenesis provide valuable clues for the identification of key signals redeployed to promote neuron regeneration of eNSPCs after SCI. Aside from the signaling pathways discussed, such as Wnt/β-catenin and MAPK, the regulation modes of other developmental signals during endogenous spinal cord regeneration remain elusive, such as BMP and sonic hedgehog (SHH) 44. The role of some signaling regulators is still ambiguous, and the conclusions obtained from different studies can be contradictory due to the different experimental designs, *e.g.*, MEK/ERK 64, 119, 156, 157. Transcriptional control works in conjunction with chromatin remodeling and epigenetic alterations under strict regional and temporal manners to promote eNSPC differentiation and self-renewal 158. Aside from adding TFs and non-coding RNAs, other manipulative means, such as histone deacetylation and DNA methylation, also deserve attention 159, as do targeted gene interventions of eNSPCs enabled by in vivo gene delivery technologies, such as non-integrating adeno-associated viral transfection and CRISPR/Cas9 genome editing approaches. Further studies are required to identify and validate novel neurogenic regulators, as well as unraveling the underlying regulatory mechanism 160, 161.

## Combined treatment strategies regulate eNSPCs to promote endogenous neurogenesis after SCI

The inadequate intrinsic neuronal differentiation capacity of eNSPCs and the intricate detrimental traumatic ambient elements, *e.g.* over-reactive immuno-responses, inhibitory factors, and tissue structure damage, *etc.*, pose difficulties for strategies targeting only one single aspect of SCI. Combining strategies, which benefit from the swift development of biocompatible materials, may synergistically address these multifaceted challenges and achieve impressive remediation outcomes in both rodent and canine models (**Table 1).**

Early attempts at combined SCI treatments can be traced back to the report from Nakafuku's group published in 2006. They confirmed the better collective performance of growth factor therapy and genetic manipulation *via* local administration to promote neurogenesis and oligodendrogenesis of eNSPCs in complete transected rat models 162. Scaffold implantation exhibits its own superiority over direct local administration in controlled drug release and structural support and has become a popular method in SCI rehabilitation 43, 103. Collagen, as a major extracellular matrix component, is one of the most commonly used naturally extracted scaffold materials 169. Dai's group accomplished a series of works based on functionalized collagen for SCI restoration by eNSPCs. Several neurogenesis-promoting factors, including small molecular drugs 76, 163, exosomes 91, and EGFR signaling antagonists 164, can be codelivered selectively to the lesion site through their grafting to linearly ordered collagen. Aside from these conventional elements, their team examined SMAD signal inhibitors, WNT signal agonists, and molecules that are critical for the direct reprogramming of fibroblasts or astrocytes into neurons and the induction of NSPC neurogenesis. The results showed that the addition of four small molecules, LDN193189, SB431542, CHIR99021 and P7C3-A20, into the collagen hydrogel stimulated neuronal differentiation and restricted astrogliogenesis of eNSPCs at the lesion site, resulting in some recovery of locomotion 165.

Porous collagen scaffolds can also be modified using MAM inhibitors (EphA4LBD to inhibit ephrinB3, PlexinB1LBD to inhibit sema4D, and NEP1-40 to inhibit Nogo-66) and collagen-binding neurotrophic factors (BDNF and NT3). To realize powerful neural regrowth and motor function recovery, a comprehensive combinatorial therapy that includes functionalized scaffold implantation and local cAMP administration was created 163. Using the combined processes of electrospinning and electrohydrodynamic jet printing, aligned collagen-fibrin fibrous hydrogels with stretchy characteristics and sticky behavior have recently been created. The hydrogels can maintain intimate contact with the transected stumps, allow for the spatiotemporal release of SDF and PTX, and activate endogenous eNSPCs for SCI healing 76.

As a denatured form of collagen, gelatin is an attractive option for the construction of inserted repair materials as well 170, 171. The mechanical and functional properties of gelatin are easy to modulate through photocrosslinking, and the 3D gelatin scaffold possesses an immunomodulatory ability to a certain extent 141, 172. Impregnation with anti-inflammatory cytokines or colony-stimulating factor 1 receptor inhibitors can further strengthen the anti-inflammatory effects of the gelatin scaffold 133. In a hemisected SCI mouse model, Fan *et al.* created dual-network electroconductive hydrogels made of photocrosslinkable gelatin methacrylate hydrogels and PPy hydrogels, which led to considerable functional recovery at an early stage 167. Multifunctional hydrogels were doped with BMSC-Exos to suppress the inflammation aggravation potential of PPy.

Aside from naturally obtained base materials, biocompatible synthetic materials are promising candidates for the manufacture of scaffolds owing to their biodegradability, designable mechanical behaviors, cost-effectiveness, *etc.* Xie *et al.* developed a magnesium oxide (MgO)/poly(L-lactide-co-caprolactone) scaffold loaded with PUR and RA, and investigated its feasibility in SCI mice 168. Their group created a nanofiber hydrogel doped with multiple growth factors (NGF, BDNF, NT3, IGF-1, bFGF, EGF, CNTF, *etc.*). Newborn neurons from eNSPCs form interconnections with severed descending corticospinal tracts, which improve the BBB scoring and motor-evoked potential results of SCI rats 30. Given the electro-sensitive character of neurons, electrical stimulation has become a novel method to facilitate neural reorganization through eNSPC activation, rendering the conductive biomaterials-based repairing platforms a linchpin for the synergy between regenerative medicine and rehabilitation approaches. To create a thermosensitive electroactive hydrogel, Liu *et al.* grafted tetraniline onto a previously synthesized poly-(ethylene glycol)-co-polyvaline polymer (TPEH). They found that, when combined with functional electrical stimulation, TPEH supplemented with NGF hold therapeutic promise for treating SCI 166.

Due to the existence of the BSCB, systematic drug delivery methods are rarely employed for SCI repair 53, 173. Nanodrug delivery systems are an effective approach for altering the *in vivo* biological distribution of drugs and have recently been used for synergistic therapy of SCI. Minocycline-loaded octadecylamine-modified polysialic acid micelles specifically accumulated in the lesion site of SCI rats after intravenous injection, and minocycline maintained a long and stable medicinal efficacy for up to 75 h 126.

## Summary and outlook

The benefits of eNSPC-based therapies consist of tissue integrity preservation and autologous stem cell-derived neuronal relay formation. By virtue of the strategies reviewed above, the stemness of eNSPCs has been employed to form functional neural networks, which interconnect severed ascending and descending axons, resulting in locomotion and sensory behavioral recovery in SCI models ranging from rodents to large animals. Despite the advancements, there is still an ongoing debate regarding suitable sources of eNSPCs in the adult spinal cord, and the critical regulators that modulate their dynamic response after injury remain to be elucidated. The heterogeneity within the eNSPC pool is another crucial issue that needs to be addressed. The eNSPCs are heterogeneous at multiple levels from localization, phenotype, and functionality to the stem cell “state” (*i.e.*, quiescent vs. activated) 27, 31, 174, 175. Sound knowledge of their basic mechanisms can facilitate the fine manipulation of eNSPCs' behaviors, such as directing the differentiation of eNSPCs toward various cell types (mainly neurons, astrocytes, and oligodendrocytes) at a suitable ratio for better reconstruction. Novel approaches, including omics technologies, single-cell sequencing, and lineage tracing, will provide valuable clues to understanding eNSPCs and the intricate microenvironment in which they reside. For instance, the capacity of anamniotes (amphibians and fishes) to regenerate spinal cord tissue after injury in situ 44 has inspired investigators to interrogate the regulatory genes that activate their spinal ependymal progenitor cells to produce neurons *via* advanced sequencing means.

Acute trauma initiates a cascade of events, from excitatory amino acid release, cellular ion imbalance, and mitochondrial dysfunction to the irreversible loss of neurons and axons, myelin necrosis, immune and inflammatory responses, glial scar development, vascular damage, and high oxidative stress at the site of the lesion 3, 18. The diverse nature of SCI pathophysiology leaves little chance for a single intervention to cure all forms of SCI. Multifaceted and combinatorial methods offer feasible practical solutions by simultaneously evoking the intrinsic regeneration mechanism of eNSPCs and providing a permissive environment for enhancing endogenous repair. Beyond this, rehabilitation programs such as locomotor training 176 and spatiotemporal epidural electrical stimulation have proven their clinical efficacy following incomplete SCI and/or complete paralysis 177, 178. Incorporation of rehabilitation is necessary for full function return. However, combination therapies do not guarantee synergistic effects. Typically, simultaneous application of plasticity-enhancing therapies, *i.e.*, anti-Nogo-A antibody administration and task-specific training resulted in interference and inconsistent movement patterns in partial SCI rats 179, while extensive locomotor training following the sequential anti-Nogo-A administration in time led to a greater recovery of lost locomotor abilities 180. In addition, only a reasonable drug dosage can fully express the positive interactive effects, since overexpression can result in adverse effects 181. Optimizing the treatment timing, drug dosage and delivery manner, and rehabilitation 182 can be very useful. Moreover, age-related regenerative decline, stress-induced proliferation suppression, gender, and other elements associated with regeneration are all concerns that should be considered when developing synchronous combined treatments. Finally, it is difficult to trace the causes of both the successes and failures of combination therapies. Thus, reports of typical failure results may also be informative and cost-efficient for the field.

## Figures and Tables

**Table 1:** Combination strategies regulate eNSPCs to promote endogenous neurogenesis after SCI

Reference	Injure model	Combination strategy	Delivery method	Outcome
Ohori *et al.*, 2006^162^	T10 spinal cord complete transection rat model	EGF, FGF2, and overexpression of Ngn2	Direct administration to injured tissue	Led to a large number of mature newborn neurons.
Li *et al.*, 2016^163^	T10 spinal cord complete transection rat model	MAIs inhibitors (EphA4LBD, PlexinB1LBD, NEP1-40), BDNF, and NT-3	Implantation of a LOCS	The full combinatorial therapy exhibited the greatest advantage for reducing the cavitation volumes, facilitating axonal regeneration, neuronal regeneration, revascularization and enhancing locomotion recovery.
		cAMP (Promotes axon regeneration)	Direct administration to uninjured tissue	
Fan *et al.*, 2018^164^	T10 spinal cord complete transection rat model	Cetuximab and PTX	Implantation of a LOCS	Cetuximab inhibited EGFR signaling, which was activated by MAIs, and PTX promoted neuronal differentiation of eNSPCs. The scaffold significantly increased endogenous neurogenesis and reconstructed the neural network in a 4-mm section of excised spinal cord tissue.
Wang *et al.*, 2019^126^	T10 spinal cord contusion rat model	Polysialic acid (PSA) and minocycline (MC)	Intravenous injection of a PSA nanodrug delivery system	PSA promoted NSPC migration, axon path finding, and synaptic remodeling. Minocycline improved neuroinflammation. The combination promoted the regeneration of neurons and the extension of long axons throughout the glial scar, thereby largely improving the locomotor function of SCI rats.
Ma *et al.*, 2020^133^	T7-8 spinal cord complete transection mice model	PLX3397 and gelatin hydrogel	Implantation of a gelatin hydrogel	The combination strategy replaced the prolonged, activated microglia/macrophages *via* cell depletion and repopulation. The improved microenvironment led to the promotion of eNSPC neurogenesis and improvement of functional recovery.
Liu *et al.*, 2020^30^	T9 spinal cord complete transection rat model	Regeneration factor “cocktail” (BDNF, bFGF, NT-3, IGF, GDNF, β-NGF, CNTF, aFGF, EGF, PDGF-AA)	Implantation of a self-assembling peptide (F-SAP) nanofiber hydrogel scaffold	Facilitated eNSPC proliferation, neuronal differentiation, maturation, and myelination, and formed interconnection with severed descending corticospinal tracts.
Chen *et al.*, 2021^76^	T8 spinal cord complete transection rat model	PTX and “middle-to-bilateral” gradient release of SDF1α	Implantation of an aligned collagen-fibrin (Col-FB) fibrous hydrogel	The “middle-to-bilateral” gradient delivery of SDF1α directed eNSPC migration from the stumps of a transected nerve toward the defect site, and PTX promoted neuronal differentiation of eNSPCs.
Zhang *et al.*, 2021^91^	T8 spinal cord complete transection rat model	Exosomes extracted from human umbilical cord-derived mesenchymal stem cells (MExos) and PTX	Implantation of a LOCS	MExos promoted the migration of eNSPCs, LOCS to retain eNSPCs, and PTX directed eNSPC differentiation into more neurons. The multifunctional scaffold showed the ability for promotion of motor functional recovery by enhancing neurogenesis and reducing glial scarring.
Yang *et al.*, 2021^165^	T8 spinal cord complete transection mice model	LDN193189, SB431542, CHIR99021 and P7C3-A20	Administration of an injectable collagen hydrogel	The combination of small molecules doped with collagen hydrogel promoted migration, induced neurogenesis and inhibited astrogliogenesis of eNSPCs in the injury site, leading to a small recovery of locomotion.
Liu *et al.*, 2021^166^	T10 spinal cord hemisecting transection rat model	NGF, soft thermosensitive polymer electroactive hydrogel (TPEH), and functional electrical stimulation (ES)	Implantation of a TPEH	NGF, electroactive hydrogel, and ES can all induce endogenous neurogenesis. Their combination provided powerful stimulation for eNSPCs to differentiate into neurons, resulting in effective functional repair.
Zhu *et al.*, 2021^144^	T8-9 spinal cord complete transection mice model	Mg/Al layered double hydroxide (Mg/Al-LDH) nanoparticles and NT-3	Implant the LDH clay biomaterials	LDH possess a great property to improve inflammatory environment and NT-3 provide the nutritional support for eNSPCs neurogenesis. The combination of them exhibited more new-born neurons in lesion core and better locomotor function recovery than LDH itself.
Fan *et al.*, 2022^167^	T9-10 spinal cord hemisecting transection mice model	BMSC-Exo and electroconductive hydrogels	Implantation of an electroconductive hydrogel composed of photocrosslinkable gelatin methacrylate hydrogels and PPy hydrogels	BMSC-Exo can reduce inflammation induced by electroconductive hydrogel. In addition, the combination strategy significantly decreased the number of CD68-positive microglia and enhanced eNSPC recruitment and neuronal differentiation, resulting in significant functional recovery.
Xie *et al.*, 2022^168^	T9 spinal cord hemisecting transection mice model	Purmorphamine (PUR, a SHH signaling agonist), retinoic acid (RA), and magnesium oxide (MgO)/poly(L-lactide-co-ε-caprolactone) (PLCL) scaffold	Implantation of a PLCL scaffold	SHH and RA induced eNSPCs recruitment and neuronal differentiation. PLCL released Mg^2+^ to promote cell survival by blocking the calcium influx. The combination strategy led to the recovery of locomotor function of SCI mice.
